# Advances in Green Nanotechnology for Water Treatment: A Systematic Review of Uranium and Thorium Removal from Water

**DOI:** 10.3390/nano16130807

**Published:** 2026-06-30

**Authors:** Simão Martins, Maria de Lurdes Dinis, Beatriz Bento, Maria Cristina Vila, Erika Andrea Levei, Anamaria Iulia Török, Deniz Avsar, Mila Kristiina Pelkonen, Niroshan Gajendra, Laura Ferrando-Climent

**Affiliations:** 1 CERENA, Faculty of Engineering, University of Porto, 4200-465 Porto, Portugal; up201905371@edu.fe.up.pt (S.M.); bbento@fe.up.pt (B.B.); mvila@fe.up.pt (M.C.V.); 2 Research Institute for Analytical Instrumentation Subsidiary, National Institute of Research and Development for Optoelectronics, INOE 2000, 67 Donath Street, 400293 Cluj-Napoca, Romania; erika.levei@icia.ro (E.A.L.); iulia.torok@icia.ro (A.I.T.); 3 Department of Tracer Technology, Environmental Technology Section, Institute for Energy Technology, 2027 Kjeller, Norway; deniz.avsar@ife.no (D.A.); niroshan.gajendra@ife.no (N.G.); laura.ferrando-climent@ife.no (L.F.-C.); 4 Department of Environmental Safety and Radiation Protection, Environmental Technology Section, Institute for Energy Technology, 2027 Kjeller, Norway; mila.pelkonen@ife.no

**Keywords:** green nanoparticles, green synthesis, uranium removal, thorium removal, water treatment, nanoparticles

## Abstract

This systematic review evaluates the application of green-synthesized nanoparticles for the removal of uranium (U) and thorium (Th) from contaminated water sources. The study focuses on the synthesis methods, adsorption performance, operational parameters, and environmental implications associated with these nanomaterials. Following PRISMA guidelines, studies published between 2015 and 2025 were identified through searches conducted in the Scopus and Web of Science databases. The review highlights the predominance of iron-based nanoparticles, biochar-derived materials, and biopolymer-based composites, many of which demonstrated removal efficiencies exceeding 90% and high adsorption capacities for U (VI) and Th (IV). Key factors influencing performance include solution pH, adsorbent dosage, contaminant concentration, and contact time. The review also examines adsorption mechanisms, regeneration potential, scalability challenges, and concerns related to environmental safety and nanoparticle recovery. Although the reported results demonstrate significant potential for radionuclide remediation, most studies remain limited to laboratory-scale experiments using synthetic water. This review synthesizes current knowledge, identifies existing research gaps, and discusses future directions required to support the practical implementation of green nanotechnologies for sustainable water treatment.

## 1. Introduction

The contamination of water resources by heavy metals and radionuclides presents a significant threat to the environment and public health, prompting an increasing demand for effective and sustainable treatment methods [1,2]. Among the most hazardous pollutants found in water are naturally occurring radioactive materials (NORM), with uranium (U) and thorium (Th) being particularly concerning [3,4]. Uranium, aside from its radioactive properties, is chemically toxic, possesses an extremely long half-life, and causes irreversible biotoxicity in various organisms [5,6]. Recognizing these risks, the World Health Organization (WHO) has set the maximum allowable concentration of U in drinking water at 30 µg/L [7]. Human activities such as uranium mining, phosphate processing, and oil and gas drilling are known to increase radionuclide levels in soil and water, thereby compounding the risk of contamination [8,9].

Rare earth elements (REEs) frequently coexist with naturally occurring radionuclides in REE-bearing minerals such as monazite, xenotime, and apatite [4]. As a result, U and Th can concentrate on tailings and wastewater generated during the mineral processing of REE ores. This accumulation represents a considerable contamination risk if not managed appropriately, as both elements are persistent contaminants that may pose radiological and toxicological risks depending on their concentration, speciation, and exposure pathway [3]. In particular, uranium is of high concern due to its greater mobility under oxidizing aqueous conditions, which increases the risk of transport to groundwater and surrounding water bodies [10], while Th tends to remain in solid residues and process streams associated with REE beneficiation [11]. Several incidents have been documented in regions impacted by REE mining and processing, where elevated U concentrations have been identified in groundwater [12,13]. These occurrences highlight the environmental and radiological hazards associated with poor residue management and the weathering of REE-containing materials [11]. Therefore, the development of effective and sustainable treatment strategies for the specific removal of uranium and thorium from contaminated waters is essential to reduce long-term environmental risks and support safer mineral resource production.

Traditionally, the treatment of contaminated water—including wastewater containing heavy metals and radionuclides—has relied on established techniques such as ion exchange, membrane processes, precipitation, solvent extraction, filtration, and conventional adsorption [10,14]. While these approaches have demonstrated effectiveness, they are often constrained by practical and economic limitations, particularly when implemented on a large scale [15,16].

A significant drawback of many traditional synthesis and treatment methods is their requirement for high energy, which increases operational costs. This issue is evident, for instance, in the production of carbon-based adsorbents, where the preparation process is notably expensive [15]. Furthermore, conventional chemical processes often rely on toxic and hazardous chemicals, volatile organic solvents, and sophisticated synthesis equipment, adding complexity and environmental risks to the treatment process [17,18].

To address the challenges posed by traditional treatment methods, nanomaterial-based strategies have gained prominence, particularly through the utilization of particles with dimensions ranging from 1 to 100 nm. These nanoparticles offer unique advantages, most notably a high surface area-to-volume ratio, which enhances their effectiveness and sustainability as treatment options for contaminated water sources [2,19].

A key advancement in this field is the concept of green synthesis, which represents a central component of green nanotechnology applied to water treatment. Green nanotechnology is understood as the development of nanoscale materials produced through environmentally oriented synthesis routes, including the use of plant extracts, microorganisms, biopolymers, biomass-derived precursors, and waste-based resources as reducing, stabilizing, capping or supporting agents [17]. Unlike conventional chemical synthesis routes, green synthesis presents numerous benefits. It is non-toxic, pollution-free, environmentally friendly, and more cost-effective [20]. This green synthesis leverages raw materials from plants—such as leaves, fruits, and peels—or from microorganisms, including fungi, algae, and bacteria. Additionally, waste biomass and industrial byproducts are utilized, further contributing to sustainability. The process itself eliminates the need for complex, expensive equipment, high energy consumption, and hazardous chemicals [18].

As a result, green synthesis plays a crucial role in developing advanced adsorbents with high performance, capable of efficiently removing radionuclides, such as uranium, from contaminated water [21].

Despite the well-established initial effectiveness of green-synthesized nanoparticles for heavy metal removal, several significant challenges impede their large-scale practical application. Most current research remains focused on laboratory or bench-level studies, leaving critical gaps in understanding how these materials can be produced and utilized at an industrial or field scale. Key issues include the scalability of nanoparticle production processes and the long-term stability of these materials under complex environmental conditions. Additionally, questions persist regarding the cost-effectiveness of deploying green-synthesized nanoparticles in mass treatment scenarios, which is essential for their adoption in real-world settings [2,22].

Another major concern is insufficient knowledge of the behaviour and potential toxicity of green-synthesized nanoparticles, particularly when they are not properly disposed of after use. This lack of understanding could have implications for environmental safety and public health, emphasizing the need for a thorough investigation into the fate and impact of these materials beyond their initial application [4]. Given these challenges, this systematic review aims to identify and synthesize available studies on green nanoparticles for U and Th removal from water, integrating information on their synthesis methods, green agents, material properties, adsorption performance, operational conditions, environmental safety, and implementation challenges. By doing so, it provides a focused overview of the current state of this emerging field and identifies the main gaps that need to be addressed before these materials can be considered for real-world radionuclide water treatment.

## 2. Materials and Methods

### 2.1. Protocol and Registration

**Figure 1:**
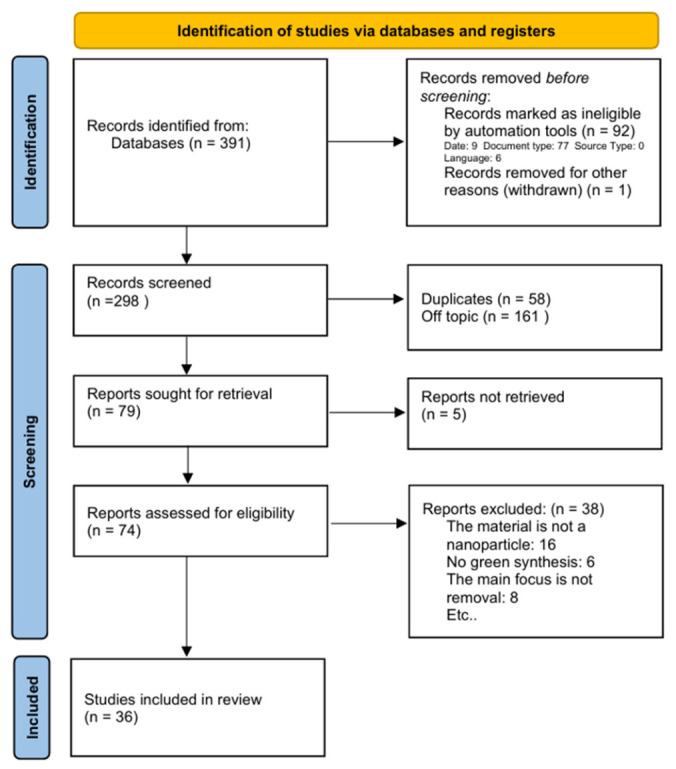
Prisma flow diagram illustrates the identification, screening, eligibility assessment, and final inclusion of studies in the systematic review.

This systematic review was conducted in accordance with the Preferred Reporting Items for Systematic Reviews and Meta-Analyses (PRISMA) guidelines (Supplementary Material), which provide a structured framework for conducting and reporting systematic reviews to ensure transparency, reproducibility, and methodological rigor throughout the review process [23]. Reporting followed the PRISMA 2020 guidance, and the study selection process is presented using a PRISMA flow diagram (Figure 1). A protocol was developed a priori; however, it was not registered in an external registry.

### 2.2. Search Strategy

This research was conducted in August 2025. Two databases were used to conduct this study: Scopus and Web of Science. Keywords related to the research topic were identified and structured into four main conceptual groups. Group 1 included terms describing the synthesis’s sustainable or biological nature, such as “green”, “biosynthesis”, “biogenic synthesis”, “waste-based”, “biogenic”, and “sustainable”. Group 2 contained terms identifying the material type, including “nanoparticles”, “nanomaterials”, and “nanosorbent”. Group 3 focused on the target contaminants, specifically “uranium”, “thorium”, and “radionuclides”. Finally, Group 4 included terms related to the application and process, such as “removal”, “adsorption”, “water”, and “water treatment”. Within each group, keywords were combined using the Boolean operator OR, and the four groups were then connected using the Boolean operator AND.

For the Scopus database, the search was conducted through the following query, inserted in Title/Abstract/Keywords:

TITLE-ABS-KEY ((“green” OR “biosynthesis” OR “biogenic synthesis” OR “waste based” OR “biogenic” OR “sustainable”) AND (“nanoparticles” OR “nanomaterials” OR “nanosorbent”) AND (“uranium” OR “thorium” OR “radionuclides”) AND (“removal” OR “adsorption” OR “water” OR “mine water treatment” OR “water treatment”)).

The Web of Science database was searched through the “Topic” field, which includes the title, abstract, and keywords.

In the records identification phase, both automatic and manual methods were used to filter the information, helping identify ineligible records before proceeding to the subsequent screening stages.

### 2.3. Eligibility Criteria

To ensure the relevance and quality of the selected literature, a rigorous set of eligibility criteria was developed and applied during the screening process. For a study to be considered, it was required to investigate the removal or adsorption of uranium (U) and/or thorium (Th) from an aqueous medium. Furthermore, the research had to describe the synthesis and application of a nanomaterial produced via a demonstrably green method. In this review, “green synthesis” was defined as the use of plant extracts, microorganisms, biopolymers, biomass-derived precursors, waste-derived materials, or other low-toxicity natural agents as reducing, stabilizing, capping, templating, or supporting components during nanomaterial production. Studies were also considered when the authors explicitly described the synthesis route as “green synthesis”, “biosynthesis”, “biogenic synthesis” or a waste-derived sustainable process. However, because the literature does not apply a uniform quantitative standard for greenness, this classification was based on the synthesis route reported by the studies rather than on a formal “greenness score”. The possible use of auxiliary chemical reagents, energy-intensive steps, or post-synthesis functionalization was therefore considered when interpreting the environmental relevance of each material, rather than being used as an automatic exclusion criterion. Finally, the application context was strictly limited to water treatment.

Conversely, several criteria were established for exclusion. The search was limited to peer-reviewed journal articles published in English between 2015 and 2025. Studies were also excluded if the full text could not be retrieved or if the publication was not an original research article (e.g., reviews or conference proceedings). Thematically, publications were also excluded if their primary focus was on contaminant detection, analysis, or remobilization rather than removal.

### 2.4. Study Selection and Data Extraction

The study selection process was conducted independently by two researchers. Initially, the titles and abstracts of all records identified through the database search were reviewed to determine if they met the established inclusion criteria. Zotero, a reference management software, was used to identify and remove duplicate studies. Articles that passed the initial screening were subjected to a full-text review for final eligibility assessment. Any discrepancies between the reviewers regarding the inclusion of a study were resolved through discussion until a consensus was reached.

Following the final selection, the two researchers independently extracted data from the included studies and tabulated them in a Microsoft Excel spreadsheet. The following information was extracted from each study: title; author; publication year; country; synthesis method; green agent used; material name; material size; specific surface area; target contaminant; water source; maximum adsorption capacity; removal efficiency (%); equilibrium time; and the key factors tested.

### 2.5. Quality Assessment

The quality and risk of bias (ROB) of each included study were independently assessed by two reviewers. Any disagreements that arose during this process were resolved through discussion to reach a consensus, with a third reviewer mediating if necessary.

Acknowledging the absence of a universally standardized ROB tool for in vitro experimental studies, a custom assessment tool was developed a priori based on the domain-based principles outlined in the Cochrane Handbook [24]. This evaluation focused on five key domains designed to capture the most relevant sources of bias in this research field: (1) Control Group, (2) Methodological Design, (3) Material Characterization, (4) Evaluation Methodology, and (5) Clarity of Results & Conclusions.

For each domain, a judgment was assigned as “Low,” “Some concerns,” or “High” risk of bias. The overall ROB for each study was then determined based on these domain-level assessments: a study was rated as “High risk” if it had a high risk in at least one critical domain; “Unclear risk” if it raised some concerns in at least one domain but had no high risks; and “Low risk” only if all domains were rated as low risk.

The ROB assessment was used as a structured appraisal framework to support the interpretation of study quality and evidence robustness; no study was excluded solely based on the ROB classification.

## 3. Results

### 3.1. Study Selection

The study selection process, guided by the PRISMA methodology, is illustrated in Figure 1. The initial database search identified 391 records. Prior to the main screening, 93 records were removed either by automated tools (period, document type, source, and language). The remaining 298 records were then screened, leading to the exclusion of 58 duplicates and 161 articles based on title and abstract.

Subsequently, 79 reports were sought for full-text retrieval, of which 5 were unavailable. This left 74 reports for eligibility assessment. After a full-text analysis, 38 of these reports were excluded for not meeting the inclusion criteria (e.g., the material was not a nanoparticle, the focus was not removal). Ultimately, 36 studies were included in the review.

### 3.2. Study Charachteristics

**Figure 2:**
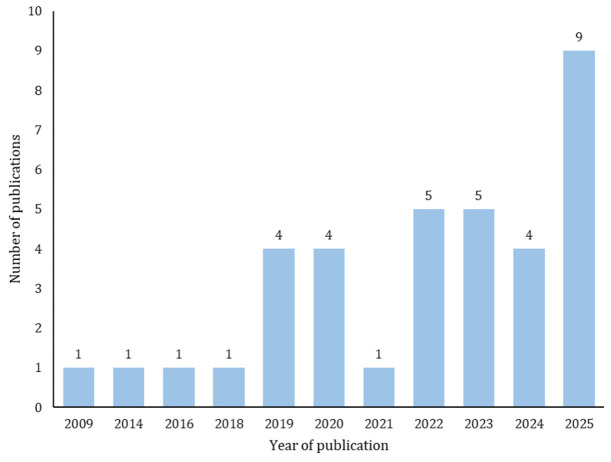
Distribution of included studies by publication year.

The distribution of publications by year is illustrated in Figure 2. The selected articles were published between 2009 and 2025, with a notable increase in research output since 2019.

**Figure 3:**
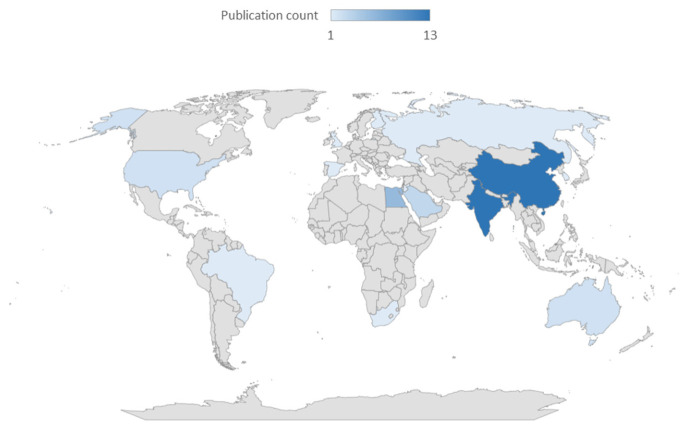
Geographical distribution of the included studies.

The geographical distribution of the included studies is presented in Figure 3. The analysis shows that research in this field, while globally distributed, is predominantly led by institutions in Asia. Specifically, China (*n* = 13) and India (*n* = 13) are the most prolific countries, accounting for most publications. Other active countries include Egypt, Saudi Arabia, and Brazil.

**Figure 4:**
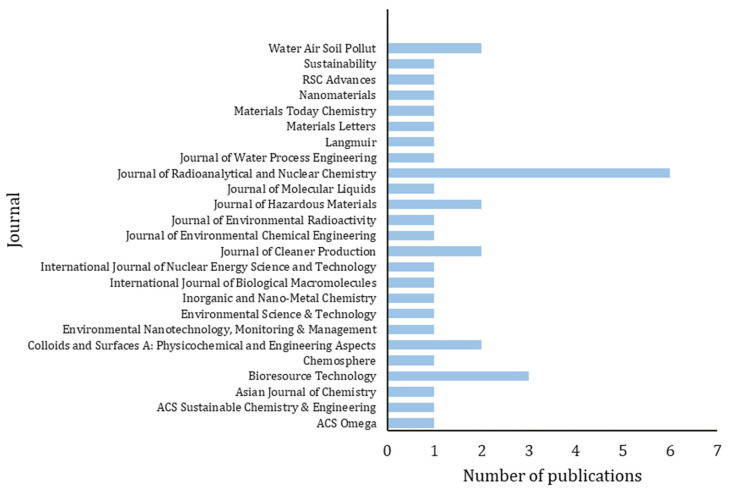
Main publication sources for the included studies.

Figure 4 illustrates the sources of the included articles detailing the specific journals from which they are derived.

**Figure 5:**
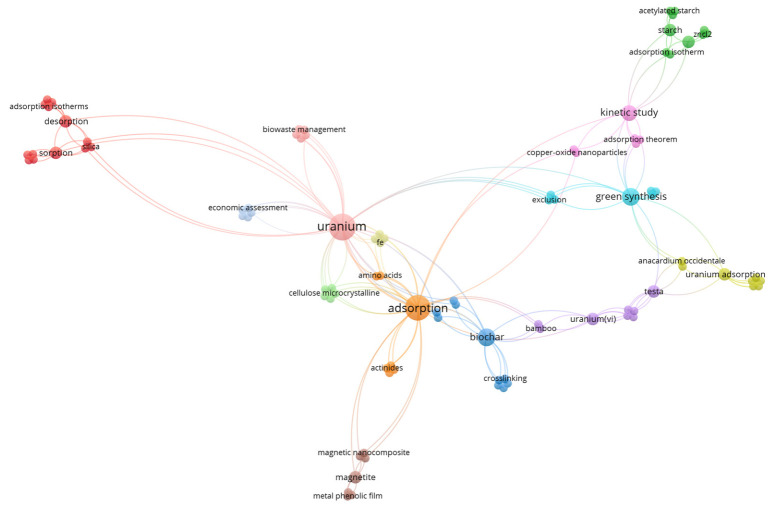
Co-occurrence network of keywords from the included studies.

Furthermore, a keyword co-occurrence analysis was generated using VOSviewer software, version 1.6.20, as shown in Figure 5, to identify the main research themes. The analysis reveals that “adsorption” and “uranium” are the central concepts connecting the main research themes. A significant research stream, highlighted by the green cluster, focuses on the application of “green synthesis” to develop novel adsorbent materials, a topic closely linked to “kinetic studies”. Furthermore, the red cluster indicates a distinct research area centered on the fundamental mechanisms of the process, grouping key terms such as “adsorption isotherms” and “desorption”.

### 3.3. Data Synthesis Strategy

Due to heterogeneity in study designs, a narrative synthesis was conducted, with descriptive statistics used where appropriate. This approach was chosen because the significant methodological differences among the studies made it unfeasible to pool the results for a meta-analysis. To ensure clarity, the findings were organized into the following categories: nanomaterial type, green synthesis agent, synthesis method, material size, surface area, target contaminants, maximum adsorption capacity, removal efficiency, equilibrium time, and key experimental factors tested.

Information that was missing from the original reports was marked as NR (not reported), and data that was not clearly presented by the authors was labeled as “unclear” (Table 1 and Table 2).

### 3.4. Types of Green Nanoparticles and Synthesis Methods

The analysis of the 36 included articles reveals a primary focus on U removal. A smaller subset of six studies addresses the adsorption of Th, either individually or in combination with uranium. The green synthesis of nanomaterials for this purpose is characterized by a notable prevalence of iron-based materials, particularly iron oxide (Fe_3_O_4_) nanoparticles and nano zerovalent iron (nZVI). These materials were explored in various configurations, including Fe_3_O_4_ NPs modified with tannic acid [25] and phytate, as well as magnetic composites functionalized with chitosan [26], glutathione [27], and β-cyclodextrin [28]. The development of biocomposites using plant extracts, such as those from papaya bark [29] and amla bark [30], was also a common approach. Further studies focused on the immobilization of Fe_3_O_4_ NPs onto biochar derived from algae [31] and bamboo [32], or their conjugation with recombinant cyanobacterial metallothioneins [33]. The synthesis of iron NPs was mediated by extracts from *Terminalia bellirica* [34], *Tinospora cordifolia* [35], and *Anacardium occidentale* testa [36], in addition to microorganisms like the fungus *Penicillium commune* [37]. Beyond oxides, nZVI was prepared via carbothermal reduction using starch and immobilized on biochar [38], or supported on activated carbon derived from peanut shells [39].

Another significant class of nanomaterials comprises carbon-based adsorbents derived from biochar produced from waste biomass, such as rice husks [40,41] or *Liquidambar styraciflua* fruits [42]. In addition to biochar, other carbon nanomaterials were investigated, including carbon quantum dots derived from starch [43] and graphene oxide composites functionalized with silica extracted from waste materials [40].

A prominent trend identified is the use of biopolymers and waste biomass as direct adsorbents or as scaffolds. Materials such as banana peels were directly processed into nanosorbents [44], while biopolymers, including cellulose [45], lignin [46], oat starch [47], and sodium alginate [48] were employed to create advanced composites and hydrogels. The use of microorganisms, such as sulfate-reducing bacteria, was also harnessed to produce stable biohybrids for uranium treatment [49]. The synthesis was frequently mediated by green reagents, such as oleic acid [50] or plant extracts derived from pomegranate [51], lemon [52], and eucalyptus [53].

In addition to the materials mentioned above, other oxides and minerals were also explored. Zeolite–hydroxyapatite composites derived from fly ash waste [54] and bacterially mediated hydroxyapatite [55] demonstrated a high affinity for radionuclides. Nanoparticles of various metal oxides, including copper oxide (CuO) [51,56], cerium oxide (CeO_2_) [52], Zn-Al layered double oxides [53], and strontium cobaltite (SrCoOx) [50], were successfully synthesized using plant extracts. Lastly, the green synthesis of nano zerovalent copper (nZVCu) mediated by *Anacardium occidentale* extract also proved effective for uranium removal [57]. More complex approaches included the use of bioelectrochemical systems with cobalt nanoparticles [58] and the fabrication of metal–organic frameworks (MOFs) [48] and zeolitic imidazolate frameworks (ZIFs) [59].

### 3.5. Removal Efficiency and Performance Metrics

Across the reviewed studies, green-synthesized nanomaterials demonstrated remarkable performance in removing uranium and thorium. The maximum adsorption capacities (q_max) varied significantly depending on the material’s composition and experimental conditions, ranging from modest values to exceptionally high capacities, such as those reported for Zn-Al layered double oxides (1153.71 mg/g for Th(IV)) [53], phytate-coated Fe_3_O_4_ NPs (948 mg/g for U(VI)) [60], and zeolite–hydroxyapatite composites (872 mg/g for U(VI)) [54]. The majority of the articles reported high removal efficiencies, consistently exceeding 90% and often approaching 99% under optimal conditions [34,39,47,48]. Adsorption kinetics also showed considerable variation. While many systems required several hours to reach equilibrium [32,49,55], a notable number of adsorbents exhibited ultra-fast performance, achieving equilibrium in minutes or even seconds [35,42,43,45,53]. Furthermore, the potential for practical application was underscored in multiple studies that successfully demonstrated the materials’ stability and high removal efficiency over several adsorption–desorption cycles, confirming their potential for regeneration and reuse [27,41,49,54].

The performance of the green-synthesized nanomaterials was evaluated across a range of physicochemical parameters to determine optimal adsorption conditions and elucidate the underlying removal mechanisms. A near-universal factor across studies was solution pH, consistently identified as the most critical parameter governing adsorption. Other key operational parameters extensively studied include adsorbent dosage, initial contaminant concentration, and contact time, which are fundamental for defining the system’s capacity and kinetics. The influence of temperature was also frequently assessed to determine the thermodynamic nature of adsorption, often revealing spontaneous, endothermic processes. Finally, to evaluate the robustness and selectivity of the nanomaterials for real-world applications, the effect of coexisting ions was a common focus. Many adsorbents demonstrated high performance even in complex water matrices, such as phosphorylated-cellulose nanocrystal ferrihydrite, which maintained over 88% removal efficiency for U(VI) in the presence of a wide array of competing cations and anions [45].

### 3.6. Risk of Bias Within Studies

**Figure 6:**
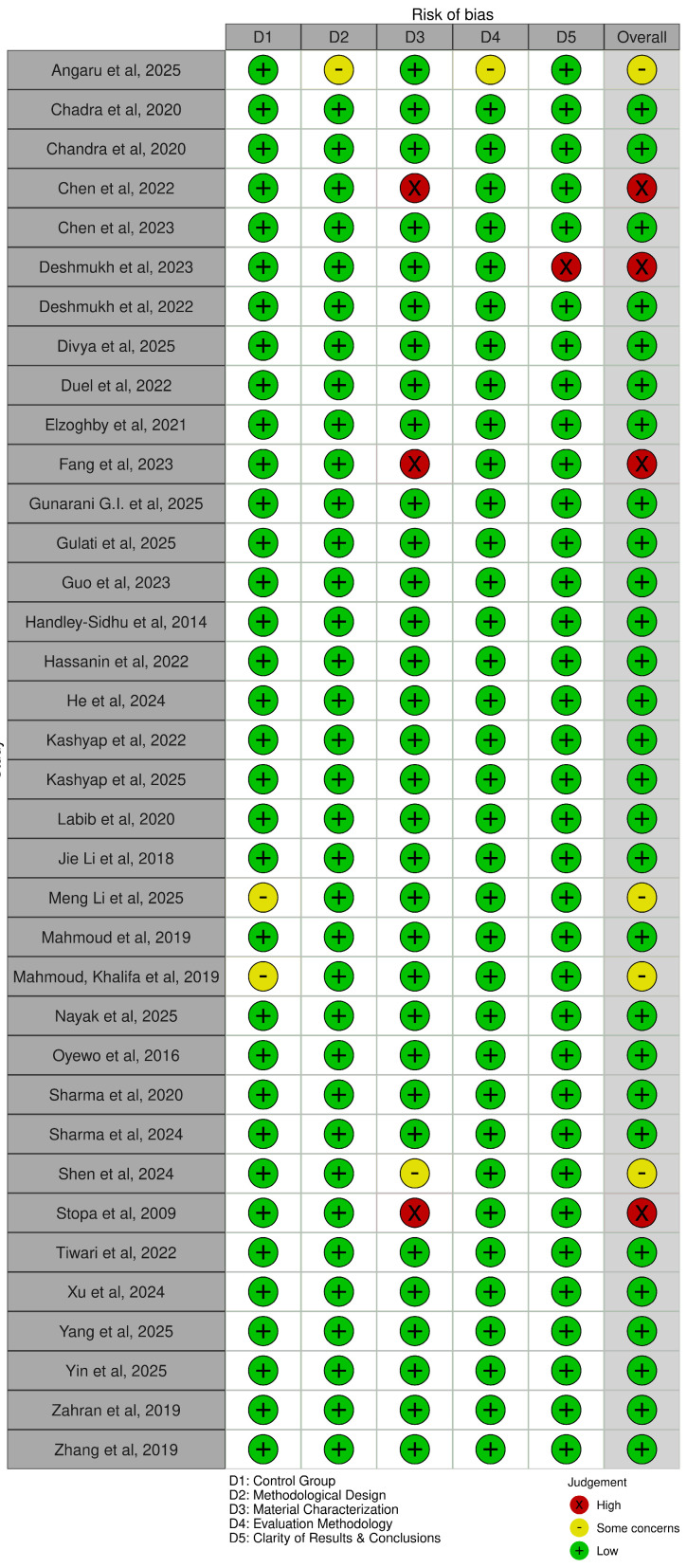
Risk of Bias summary for the studies included in the systematic review [25,26,27,28,29,30,31,32,33,34,35,36,37,38,39,40,41,42,43,44,45,46,47,48,49,50,51,52,53,54,55,56,57,58,59,60].

**Figure 7:**
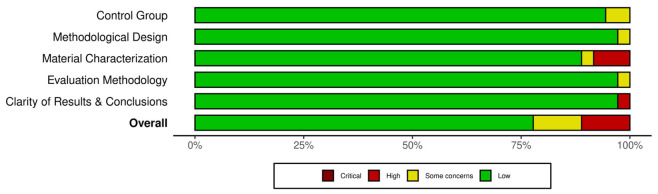
Risk of Bias graph.

The risk of bias (ROB) for each study was assessed by evaluating the clarity and methodological soundness of the following domains: control group, study design, material characterization, evaluation method, results, and conclusions. Summary figures for this assessment were then generated using the robvis visualization tool [61]. The overall analysis indicated that most studies had a low risk of bias (Figure 6 and Figure 7). However, the “Material Characterization” domain was the one that presented the highest proportion of studies rated as “high risk” or having “some concerns”. This was mainly associated with incomplete reporting of specific characterization parameters in some studies, particularly nanoparticle size and, in some cases, surface area. Although all included studies described the materials as nanomaterials or nanoparticles and therefore met the inclusion criteria, missing characterization data may limit direct comparison among materials and reduce the certainty with which adsorption performance and mechanisms can be linked to specific physicochemical properties.

**Table 1:** Characteristics of Green-Synthesized Nanomaterials for Uranium and Thorium Removal.

Study	Nanoparticle (Np)	Green Agent Used	Synthesis Method	Material Size	Surface Area
[54]	HApZ	Fly ash	One-step hydrothermal method	Unclear	301.5–351 m^2^ g^−1^
[57]	AO-Cu	*Anacardium occidentale* testa extract	Reduction in cupric chloride dihydrate with *Anacardium occidentale* testa extract, heated at 60–70 °C with stirring	<30 nm	^1^ NR
[36]	Ao-Fe	*Anacardium occidentale* testa extract	FeCl_3_·6H_2_O solution mixed with the green extract, heated at 70 °C for 15 min, centrifuged, washed and dried	70–90 nm	NR
[53]	Zn/Al-bimetallic layered double oxides (ZnAlLDO)	Eucalyptus leaf extract	Template-calcination method	NR	NR
[32]	Fe_3_O_4_@MBC	Bamboo waste	Solvothermal reaction in an organic solvent	Unclear	66.04–129.79 m^2^ g^−1^
[30]	Magnetic bio composite (Fe_3_O_4_)	Amla tree bark (*Phyllanthus emblica* Linn)	Chemical precipitation (bottom–up approach)	12.1 nm	NR
[29]	Fe_3_O_4_@PBP	Papaya bark	Co-precipitation of Fe^2+^ and Fe^3+^ using papaya bark powder (PBP) as a reducing and/or stabilizing agent, stirred at 60–70 °C for 24 h	26.4 nm	Unclear
[33]	CMNP-NmtA	Recombinant Anabaena metallothionein (NmtA), originally from cyanobacterium *Anabaena* sp. PCC 7120; Citric acid	Citric acid-functionalized magnetic nanoparticles (CMNPs) synthesized by co-precipitation of Fe^2+^ and Fe^3+^ ions, then activated by EDC-NHS coupling. Purified recombinant Anabaena NmtA protein immobilized onto activated CMNPs via amide linkage	9.3 nm	NR
[60]	Phy@Fe_3_O_4_	Phytate	One-pot single-step synthesis via vigorous stirring of iron chlorides and phytic acid sodium salt in basic aqueous medium at 25 °C for three minutes. Co-precipitation method	80–160 nm	Unclear
[51]	CrO@PA6, CuO@PA6	Pomegranate (*Punica granatum* L.) peel extract	Synthesis of chromium oxide and copper oxide nanoparticles using Pomegranate Peel Extract as a reducing and capping agent, followed by incorporation into Polyamide 6 matrices via melt compounding technique	CrO NPs: 20 nm; CuO NPs: 32 nm	CrO@PA6: 21.67 m^2^ g^−1^; CuO@PA6: 16.89 m^2^ g^−1^
[39]	nZVI/BC600	Peanut shells (for activated carbon)	Activated carbon was prepared by pyrolyzing peanut shells with ZnCl_2_. Nanoscale zerovalent iron (nZVI) was loaded onto the carbon support via liquid phase reduction in ferrous sulfate with potassium borohydride	Unclear	NR
[34]	Biogenic Fe (B-Fe), Fe/Ni (B-Fe/Ni)	*Terminalia bellirica* extract	Synthesized by mixing the green extract with aqueous iron (and nickel) salt solutions under ambient conditions, followed by ultrasonication and collection of the precipitate.	9.33–12.23 nm	B-Fe: 12.23 m^2^ g^−1^, B-Fe/Ni: 4.52 m^2^ g^−1^
[47]	Nano-starch, acetylated nano-starch	Oats	Nano-starch extracted from oats, then acetylated using acetic anhydride	86.03–189.5 nm	NR
[46]	AL-PEI	Lignin	Two-step process of synthesizing surface-functionalized lignin adsorbent (AL-PEI) with dithiocarbamate and amine functional groups by using alkaline lignin (AL), polyethylenimine (PEI), and carbon disulfide (CS2) as raw materials	21 nm	22.94 m^2^ g^−1^
[55]	Bacterially Produced Hydroxyapatite (BHAP), heat-treated BHAP (e.g., 450-BHAP, 700-BHAP)	*Serratia* sp. bacterium	Synthesized by incubating *Serratia* sp. bacteria with calcium chloride, sodium citrate, and glycerol 2-phosphate in buffered solution at 30 °C with shaking	32–271 nm	Initial-BHAP: 40 m^2^ g^−1^, 400-BHAP: 115 m^2^ g^−1^, 700-BHAP: 12 m^2^ g^−1^
[40]	SiO_2_/GO (silica/graphene oxdide)	Rice husks, spent carbon rods batteries	Silica extracted from rice husk ash via a wet chemical process. Graphene oxide is produced from waste zinc-carbon battery rods using the Hummer method. The final composite was formed by reacting to the silica with graphene oxide.	40 nm	SiO_2_: 25.89 m^2^ g^−1^, GO: 37.37 m^2^ g^−1^, SiO_2_/GO: 35.45 m^2^ g^−1^
[49]	BX-FeS	Sulfate-reducing bacteria (Desulfovibrio desulfuricans), Xanthan gum	Synthesized by incubating Desulfovibrio desulfuricans bacteria with an iron sulfate solution and xanthan gum under anaerobic conditions for three days, producing xanthan gum-stabilized biogenic mackinawite nanoparticles	FeS < 50 nm; BX-FeS: 792.6 nm	NR
[52]	CeO_2_	Citrus limon peel extract	Cerium oxide nanoparticles were synthesized by mixing an aqueous extract of Citrus limon peel with an ammonium cerium nitrate precursor solution. The mixture was heated and stirred	10 nm	NR
[56]	CuO-NPs	Flower extract of Nyctanthesarbor-tristis plant	Nyctanthes arbor-tristis flowers were mixed with a cupric acetate solution and stirred for 24 h. The resulting precipitate was then collected via centrifugation and calcinated	<30 nm	Unclear
[50]	SrCoO_x_	Oleic acid	Green chemical method using oleic acid as a green surfactant, followed by heating and thermal treatment at 400 °C (SC4) and 500 °C (SC5)	20–40 nm	SC4: 160.585 m^2^ g^−1^, SC5: 332.149 m^2^ g^−1^
[59]	PPy/ZIF-8	Polypyrrole	PPy tubes synthesized using methyl orange and FeCl_3_. Then growing ZIF-8 nanoparticles onto the nanotubes by mixing Zn(NO_3_)_2_·6H_2_O and 2-MeIM in methanol, followed by washing and drying	NR	PPy/ZIF-8: 1300 m^2^ g^−1^, ZIF-8: 1500 m^2^ g^−1^, PPy: 17 m^2^ g^−1^
[58]	CoNPs/NC	Cotton fibers	Two-step method: Dopamine chelation with Co2+ ions on cotton fiber surfaces, then in situ free radical polymerization forms polydopamine/cobalt composite layer. Modified fibers pyrolyzed at 900 °C for 2 h to produce CoNPs/NC	NR	330 m^2^ g^−1^
[43]	CQDs@PAFP	Starch	Synthesized by microwave-assisted pyrolysis of a starch-water solution to generate carbon quantum dots, followed by covalent immobilization of the CQDs onto a polyanthranilic acid–formaldehyde–phthalic acid matrix under reflux at 140 °C and subsequent isolation and drying of the nanobiosorbent.	35.21–73.11 nm	28.79 m^2^ g^−1^
[42]	BC-Gl-NSi	*Liquidambar styraciflua* fruit	*Liquidambar styraciflua* fruit ground pyrolyzed at 450 °C for 20 min to produce biochar. Nanosilica and biochar suspended in toluene, blended with glutaraldehyde, refluxed for 6 h. Cooled, filtered, washed, dried at 70 °C	17.32–36.25 nm	60.754 m^2^ g^−1^
[45]	PCNCFH, PMCCFH	Cellulose nanocrystals, Microcrystalline cellulose, Trisodium trimetaphosphate	Phosphorylation of cellulose using trisodium trimetaphosphate, followed by incorporation of ferric chloride to form a stable composite	Unclear	PCNCFH: 299.89 m^2^ g^−1^, PMCCFH: 276.71 m^2^ g^−1^
[44]	Banana peels nanosorbent (BPN)	Banana peels	Banana peels separated, cut, washed, sun-dried, crushed, screened to <65 mm, then acid and alkali treated, and mechanically milled	<25 nm	Unclear
[27]	Glutathione@magnetite	Glutathione	Magnetite nanoparticles were first synthesized via sonochemical co-precipitation of iron salts. The resulting nanoparticles were then functionalized by sonicating them with a reduced glutathione solution in a water/methanol mixture to create the final composite	Unclear	44.73 m^2^ g^−1^
[48]	MNPs-SA@Cu MOF	Sodium alginate	Co-precipitation of Fe^2+^/Fe^3+^ to form magnetite, entrapping the magnetite in sodium alginate droplets crosslinked in Cu(NO_3_)_2_ to make beads, and then growing a Cu–trimesate MOF in situ on the beads under mild hydrothermal conditions, followed by washing and drying	25 nm	13.603 m^2^ g^−1^
[31]	Humic acid-coated Fe_3_O_4_ nanoparticle-modified biochar from filamentous green algae (HA–Fe_3_O_4_/BC)	Green algae	Co-precipitation method. Biochar from filamentous green algae. FeSO_4_·7H_2_O and FeCl_3_ dissolved in deoxygenated water, then humic acid added, stirred at 60 °C. Biochar added, precipitated composites collected, filtered, washed, freeze-dried	Unclear	NR
[26]	Magnetic chitosan	Chitosan	Chemical precipitation method. Magnetite powder added to chitosan solution in acetic acid, then NaOH solution mixed for coating layer formation. Product filtered, washed and dried	NR	NR
[35]	Gilloy-shoot extract-reduced magnetic nanoparticles (GS@MNPs)	Gilloy (*Tinospora cordifolia*) shoot extract	Co-precipitation of ferric chloride and ferrous sulfate using Gilloy shoot extract as reducing and stabilizing agent, followed by NaOH addition and heating	23.17 nm	NR
[41]	Hydrogen Peroxide-Modified Magnetic Biochar (MBC), Hydrogen Peroxide-Modified Biochar (HBC), Biochar (BC)	Rice husks	Pyrolysis of rice husks to biochar. BC modified with hydrogen peroxide. HBC combined with synthesized Fe_3_O_4_ nanoparticles	50–150 nm	HBC: 57.304 m^2^ g^−1^, MBC: 195.62 m^2^ g^−1^
[25]	TA-Fe^III^@Fe_3_O_4_	Tannic acid	One-step synthesis: Tannic acid and ferric chloride solution mixed, then Fe_3_O_4_ particles added and shaken	50–100 nm diameter	NR
[37]	Biogenic iron oxide nanoparticles (FeO-NPs)	*Penicillium commune*	Fe(NO_3_)_3_·9H_2_O mixed with fungal culture filtrate (CFF), pH adjusted, stirred, incubated at 35 °C for 24 h in the dark, liquid evaporated, residue washed and calcined	12–40 nm	Unclear
[28]	β-cyclodextrin magnetic bentonite nanocomposite (βCD-FB)	β-cyclodextrin	Precipitation method. βCD-FB prepared by ion exchange method with CMCD added to FB suspension	<20 nm	NR
[38]	nZVI/BC	Starch	Carbothermal reduction process, using starch as carbon source and ferric salts at different temperatures under nitrogen atmosphere	Unclear	FeCl/C (1:4–900): 782.05 m^2^ g^−1^, FeN/C (1:4–900): 204.85 m^2^ g^−1^

^1^ NR—Not Reported.

**Table 2:** Adsorption Performance and Experimental Conditions of Green Nanomaterials for Uranium and Thorium Removal.

Study	Target Contaminant	Max Adsorption Capacity	Removal Efficiency	Equilibrium Time	Water Source	Factors Tested
[54]	Thorium, Uranium	Th(IV): 793 mg g^−1^, U(VI): 872 mg g^−1^	Th(IV): 81% (after 6 cycles), U(VI): 89% (after 6 cycles)	120 min	Synthetic aqueous solution, industrial effluent	pH, contact time, adsorbate concentration, temperature, competing ions, reusability
[57]	Uranium	129.87 mg g^−1^	96.63%	60 min	Synthetic aqueous solution	pH, adsorbent dosage, adsorbate concentration, contact time
[36]	Uranium	11.61 mg g^−1^	93–94%	60 min	Synthetic aqueous solution	pH, adsorbent dosage, adsorbate concentration, contact time
[53]	Thorium	1153.71 mg g^−1^	85% (after 5 cycles)	5 min	Synthetic aqueous solution	pH, ionic strength, contact time, initial thorium concentration, adsorbent dosage, competitive ions, reusability
[32]	Uranium	70.45 mg g^−1^	83.78% (after 3 cycles)	240 min	Synthetic aqueous solution	Adsorbent dosage, pH, contact time, initial uranium concentration, temperature
[30]	Uranium	121.95 mg g^−1^	90.80%	40 min	Synthetic aqueous solution	pH, adsorbent dosage, contact time, temperature, initial uranium concentration, particle size, reusability
[29]	Uranium	120.48 mg g^−1^	88.80%	40 min	Synthetic aqueous solution	pH, contact time, initial uranium concentration, adsorbent dosage, particle size, temperature, reusability
[33]	Cadmium, Uranium	U(VI): 43.32 mg g^−1^	U(VI): ~81%	U: 60 min	Synthetic aqueous solution	pH, contact time, competing ions, temperature
[60]	Yttrium, Strontium, Uranium	U(VI): 948 mg g^−1^	U(VI): 97%	120 min	Synthetic aqueous solution	pH, initial uranium concentration, adsorbent dosage, contact time
[51]	Uranium	CrO@PA6: 61.1 mg g^−1^; CuO@PA6: 53.5 mg g^−1^	CrO@PA6: 81.0%, CuO@PA6: 71.8%	120 min	Synthetic aqueous solution	pH, initial concentration, adsorbent dosage, temperature, reusability
[39]	Uranium	19.94 mg g^−1^	99.68%	30 min	Synthetic aqueous solution	pH, contact time, adsorbent dosage, initial concentration, competing ions
[34]	Chromium, Uranium	U(VI): B-Fe 4.97 mg g^−1^, B-Fe/Ni 11.49 mg g^−1^	U(VI): 99.7%	30 min	Synthetic aqueous solution	pH, initial contaminant concentration, adsorbent dosage
[47]	Uranium	1.48 mg g^−1^	97% (nano-starch), 99% (acetylated nano-starch)	30 min	Synthetic aqueous solution, real groundwater	pH, contact time, temperature, adsorbent dose, initial uranium concentration, reusability
[46]	Thorium, Uranium	Th(IV): 396 mg g^−1^ (alkaline), <40 mg g^−1^ (acidic) U(VI): 392 mg g^−1^ (alkaline), 332 mg g^−1^ (acidic);	~90% (after 5 cycles at ph = 11)	Th(IV): 60 min, U(VI): 150 min	Synthetic aqueous solution	pH, initial metal concentrations, HNO_3_ concentration, adsorbent dosage, contact time, competing ions, reusability
[55]	Strontium, Cobalt, Europium, Uranium	U(VI): 312 mg g^−1^	Unclear	24 h	Synthetic aqueous solutions, artificial groundwater	Heat treatment temperature, pH
[40]	Uranium	145.0 mg g^−1^	96.66%	50 min	Synthetic aqueous solution	pH, contact time, adsorbent dosage, initial uranium concentration, temperature, reusability
[49]	Uranium	658.0 mg g^−1^	97.9%	6 h	Synthetic aqueous solution, real uranium wastewater	pH, competing ions, storage time, adsorbent dosage, contact time, economic cost, reusability, initial uranium concentration
[52]	Uranium	46.2 mg g^−1^	94–96%	80 min	Synthetic aqueous solution	Initial uranium concentration, contact time, adsorbent dosage, pH
[56]	Cadmium, Chromium, Lead, Uranium	U(VI): 200 mg g^−1^	U: 88.6%	20 min	Synthetic aqueous solution	pH, contact time, adsorbent dosage, initial concentrations of metal ions
[50]	Iron, Thorium	Th(IV): 27.2 mg g^−1^	Th(IV): 99%	Unclear	Synthetic aqueous solution, real industrial process liquor	adsorbent synthesis temperature, pH, contact time, initial metal concentration, competing ions,
[59]	Uranium	534.0 mg g^−1^	99%	90 min	Synthetic aqueous solution	pH, contact time, initial uranium concentration, temperature, competing ions
[58]	Uranium	NR	95%	NR	Synthetic aqueous solution	Electrode materials, incubation conditions, applied voltage
[43]	Uranium	147.6 mg g^−1^	95.5–98.1%	10 s (microwave heating)	Synthetic aqueous solution, real water samples (tap water, seawater, wastewater) spiked with U(VI)	pH, adsorbent dosage, initial uranium concentration, competing ions, contact time, temperature, reusability
[42]	Uranium	NR	87.4%	1 min	Synthetic uranium solution, real tap water samples spiked with uranyl	pH, contact time, temperature, adsorbent dosage, initial uranium concentration, competing ions
[45]	Uranium	PCNCFH: 100 mg g^−1^ PMCCFH: 25 mg g^−1^	PCNCFH: >98% in presence of most anions, >88% in multi-ion solution.	2 min	Synthetic aqueous solution, simulated tap water/groundwater matrices	Adsorbent dosage, contact time, pH, competing ions, ionic strength, reusability
[44]	Thorium, Uranium	U(VI): 27.1 mg g^−1^ (synthetic water), 34.13 mg g^−1^ (real mine water); Th(IV): 45.5 mg g^−1^ (synthetic water), 10.10 mg g^−1^ (real mine water)	Th(IV): 99.99%, U(VI): 70%,	24 h	Synthetic aqueous solution, real mine water	pH, adsorbent dosage, initial metal concentration, temperature, type of water (synthetic water/real mine water)
[27]	Uranium	333.33 mg g^−1^	94.56%; 89.25% (after six cycles)	120 min	Synthetic aqueous solution	pH, temperature, contact time, initial uranium concentration, adsorbent dosage, reusability
[48]	Thorium, Uranium	U(VI): 454.54 mg g^−1^, Th(IV): 434.78 mg g^−1^	Th(IV): 97.7%, U(VI): 99.9%	Th(IV): 10 min, U(VI): 90 min	Synthetic aqueous solution	pH, contact time, adsorbent dosage, initial concentration, reusability
[31]	Uranium	555.56 mg g^−1^	NR	60 min	Synthetic aqueous solution	pH, reaction time, temperature, initial uranium concentration, competing ions, practical application (tap water vs. river water)
[26]	Uranium	42 mg g^−1^	NR	40 min	Synthetic aqueous solution	Initial uranium concentration, pH (desorption)
[35]	Uranium	93.54 mg g^−1^	98.23%	5 min	Synthetic aqueous solution	pH, adsorbant dosage, initial uranium concentration, contact time
[41]	Uranium	HBC: 69.50 mg g^−1^, MBC: 77.58 mg g^−1^	Unclear	50 min	Synthetic aqueous solution	pH, adsorbent dosage, contact time, reusability, initial uranium concentration
[25]	Uranium	98.2 mg g^−1^	99.89%	Unclear	Synthetic aqueous solution	pH, adsorbent dosage, temperature, initial uranium concentration
[37]	Uranium	94.9 mg g^−1^	91.7%	60 min	Synthetic aqueous solution	Adsorbent dosage, contact time, pH, competitive ions, incubation conditions (light/dark)
[28]	Uranium	305 mg g^−1^	61%	60 min	Synthetic aqueous solution	Adsorbent dosage, contact time, pH, initial uranium concentration, temperature, reusability
[38]	Uranium	34.82 mg g^−1^ (FeCl/C (1:4–900)), 55.14 mg g^−1^ (FeN/C (1:4–900))	93.1% (FeCl/C at pH 7), 94.3% (FeN/C at pH 6)	60–100 min	Synthetic aqueous solution	Nanoparticles synthesis: carbonization temperature, iron sources, ratio Fe/Starch; Adsorption: pH, adsorbent dosage, contact time, effect of oxidation

## 4. Discussion

### 4.1. Principal Findings

Green-synthesized nanoparticles have emerged as promising candidates for radionuclide remediation, although their large-scale viability has yet to be fully demonstrated offering a sustainable alternative to conventional methods. The principal finding across the 36 analyzed studies is that these approaches are not only environmentally conscious but also demonstrate exceptional performance, with many of the reviewed materials showing efficiencies exceeding 90% and, in several cases, reporting extraordinarily high maximum adsorption capacities.

A second key finding is that this “green” approach is increasingly synonymous with a circular economy. The synthesis of high-performance adsorbents was frequently achieved by leveraging “waste-to-resource” pathways, such as utilizing agricultural residues like rice husks [40,41] or plant-based extracts [51,53] as precursors, rather than relying on costly or toxic chemical reagents.

The analysis also revealed a clear trend in material choice, with a significant predominance of iron-based nanomaterials, particularly magnetic Fe_3_O_4_ and nZVI and operationally, solution pH is consistently identified as the most critical parameter governing the success of the adsorption process.

Despite these promising outcomes, there is a notable imbalance in research focus, with uranium removal being extensively investigated while significantly fewer studies address thorium. This disparity underscores a critical research gap and indicates that the current evidence for thorium remediation using green-synthesized nanoparticles remains comparatively limited.

### 4.2. Comparison with Conventional Methods

The remediation of heavy metal and radionuclide contamination demands efficient, selective, and environmentally friendly technologies. Conventional wastewater treatment methods, including chemical precipitation, solvent extraction, ion exchange, and the use of traditional adsorbents like activated carbon, are often constrained by inherent limitations, such as high implementation cost [62], the reliance on toxic reagents [17], the subsequent use of harmful solvents [62], and the resulting creation of polluting waste.

In contrast, the advent of green-synthesized NPs provides a compelling, sustainable alternative that successfully addresses these deficiencies while demonstrating superior performance. Green synthesis is recognized as an eco-friendly, simple, and cost-effective process by most studies [29,37,51]. This approach prioritizes sustainable development by utilizing readily available biological resources and waste materials, resulting in bio-adsorbents that are non-hazardous to human health and the environment and that effectively prevent the formation of secondary pollutants [30]. Furthermore, the phytochemical components in these extracts serve a dual function as reducing and capping agents, eliminating the need for the additional chemical modifications often required in conventional nanoparticle synthesis to ensure stability [29,35].

Several green-synthesized nanoparticles showed promising adsorption performance compared with selected conventional adsorbents:

Efficiency and Capacity: Novel green materials exhibit ultra-high uptake capacities and rapid kinetics. For instance, the maximum adsorption capacity of 792.82 mg/g and 333.33 mg/g for U(VI) achieved by HApZ composites [54] and glutathione-decorated magnetite [27], respectively, were higher than conventional adsorbents like certain commercial activated carbons, such as the one reported with an adsorption capacity of 28.49 mg/g [63] or commercial alumina with 78 mg/g [64]. In terms of removal percentages, materials such as biogenic Fe/Ni nanoparticles and MNPs-SA@Cu MOF composite beads achieved near-complete removal efficiencies (99.7% to 99.9%) within short contact times [34,48].

Selectivity: Critically, these materials maintain high performance even in complex water environments. High removal efficiencies for U(VI) and Th(IV) were demonstrated in complex multi-ion systems using MNPs-SA@Cu MOF composite beads [48], reinforcing their practical applicability. Specific bio-composites, such as Phytate-coated Fe_3_O_4_ NPs, demonstrated the ability to eliminate uranyl ions [60], and similarly, phosphorylated cellulose-ferrihydrite (PCNCFH) showed effective performance and selectivity despite the presence of typical competing ions in water [45].

### 4.3. Mechanistic Insights

Adsorption and surface complexation are key mechanisms, enhanced by biomolecular functional groups.

The removal of U(VI) and Th(IV) from water is not a single process but rather a combination of chemical and electrostatic interactions.

The primary mechanism, and what often dictates the speed of the entire process, is chemisorption through surface complexation, evidenced by favourable fits to the pseudo-second-order kinetic model [32,41,46,48,54]. This process frequently involves inner-sphere complexation, in which metal ions form stable chemical bonds with specific, electron-rich functional groups on the adsorbent surface [45,54].

Critical to establishing this strong chemical affinity are phosphate (-PO_4_^3−^), hydroxyl (-OH), amine/amidoxime (-NH_2_/C(=NOH)NH_2_), and carboxyl (-COOH) groups [35,41,45,46,48,54]. These groups actively participate in complexation and chelation reactions [30,41,45], which is often confirmed by spectroscopic shifts observed in FTIR spectra post-adsorption [41,48,54].

Parallel to complexation, electrostatic attraction plays a significant role, but its contribution is strongly dependent on solution pH and radionuclide speciation, particularly at optimal acidic to neutral pH levels where the radionuclides exist predominantly as cationic species (UO_2_^2+^ and Th^4+^), which are drawn to negatively charged surface sites [30,48,54]. However, this behavior can be altered in the presence of coexisting anions, such as carbonate, phosphate, sulfate, nitrate, or chloride, which may influence radionuclide speciation, compete for active adsorption sites, modify ionic strength, or promote the formation of aqueous complexes and/or insoluble precipitates. Therefore, adsorption performance observed under synthetic conditions should be interpreted cautiously when considering real water matrices. Furthermore, several successful systems incorporate ion-exchange mechanisms, notably in materials containing zeolite or hydroxyapatite, often enabling a dual-mode removal pathway [48,54,59].

Finally, redox reactivity is particularly relevant for uranium (VI) removal. A crucial mechanism involves reductive immobilization, often facilitated by redox-active iron-based nanoparticles (e.g., nZVI or Fe_3_O_4_), which can promote electron transfer and chemically convert soluble U(VI) to the highly stable and immobile U(IV) oxidation state [25,38,39,60]. It should also be noted that, under higher pH conditions, the spontaneous formation of insoluble precipitates (such as hydroxides or phosphates) also contributes to the overall radionuclide removal [44,54].

When considering these mechanisms by material family, the reviewed studies indicate that different green nanomaterials do not depend on a single removal pathway. In plant-mediated systems, such as tannic acid-modified Fe_3_O_4_ [25], pomegranate peel-derived oxide nanocomposites [51], eucalyptus-assisted Zn-Al layered double oxides [53], and plant-extract-mediated Fe or Cu nanoparticles [34,35,36,57], the organic compounds in the extracts contribute to both nanoparticle formation and the introduction of surface functional groups that help bind radionuclides. These groups can favor complexation, chelation, electrostatic interactions, and adsorption selectivity, since different groups containing oxygen and nitrogen can show different affinities toward U(VI), Th(IV), and competing ions. However, their effect varies based on the plant source, synthesis conditions, and the final surface chemistry reported in each study. In iron-based materials, the main mechanism depends strongly on the iron phase: nZVI-supported systems [38,39] are more directly related to the reductive immobilization of U(VI), while Fe_3_O_4_-based composites [25,27,29,30,31,32,60] mainly combine magnetic recovery with adsorption and surface complexation, with possible redox contribution under favorable conditions. In biopolymer-, biomass-, and biochar-based composites, including chitosan, cellulose, lignin, starch, sodium alginate, banana peel, and modified biochars [26,41,44,45,46,47,48], functional groups containing oxygen, nitrogen, sulfur, or phosphate provide active sites for radionuclide binding through complexation, chelation, ion exchange, or precipitation-related pathways. In composites that include magnetic or mineral phases, the organic matrix acts together with the inorganic component: the biomass fraction provides functional binding sites, while the magnetic phase contributes to separation, additional adsorption sites, reduce particle aggregation, or redox-mediated immobilization in selected iron-based systems. Mineral and porous systems, including zeolite–hydroxyapatite composites, bacterially produced hydroxyapatite, MOF-based beads, and ZIF-containing composites [48,54,55,59], further demonstrate the role of ion exchange, phosphate-related immobilization, surface complexation, and high surface area/porosity in improving radionuclide uptake. Therefore, the mechanisms reported in the reviewed studies should be seen as specific combinations of surface functionality, ion exchange, complexation, precipitation, magnetic separation, and, in certain iron-based systems, redox-mediated immobilization, rather than one universal adsorption pathway.

### 4.4. Environmental and Toxicological Insights

An essential aspect of green nanotechnology is ensuring that the synthesized materials pose minimal environmental risk throughout their life cycle. In the reviewed studies, however, only a limited number of articles provided a direct assessment of the environmental impact or toxicological profile of the developed nanosorbents. In most cases, environmental safety was inferred mainly from the synthesis route itself, with materials frequently described as “green”, “eco-friendly”, or “sustainable” due to the use of plant extracts, biopolymers, microorganisms, or waste-derived precursors. Although green synthesis can reduce the use of hazardous reagents, toxic solvents, and energy-intensive processes, it does not automatically guarantee that the resulting nanomaterials are non-toxic after application.

This distinction is particularly important because nanoparticles may present environmental risks that are not solely determined by their synthesis route. Their behaviour and toxicity depend on physicochemical properties such as particle size, morphology, surface charge, coating, aggregation state, dissolution, and chemical transformation in environmental media [65]. The role of green capping and stabilizing agents is particularly relevant in this context. Javed et al. [66] emphasized that capping agents stabilize the nanoparticle–medium interface and can inhibit particle aggregation or coagulation, thereby influencing physicochemical behaviour and environmental interactions. In green synthesis, plant-derived extracts contain phytochemicals, polysaccharides, proteins, and other biomolecules that may act simultaneously as reducing, capping, and stabilizing agents [67]. These organic compounds may improve colloidal stability and provide functional groups involved in radionuclide binding; however, their behaviour after environmental release remains uncertain. Once released into water, soil, or sediments, nanoparticles may undergo ageing, aggregation, surface modification, or interaction with natural organic matter and coexisting ions, which can alter their mobility, bioavailability, and toxicity [65]. Therefore, although green caping agents may reduce the use of hazardous reagents during synthesis, nanomaterials used for radionuclide removal should be evaluated not only in terms of adsorption performance, but also in long-term stability, degradation, desorption, and transformation under realistic water chemistry, before assuming that green-synthesized nanoparticles are environmentally safe after application.

Within the reviewed literature, some studies explicitly reported favourable safety-related characteristics. For example, iron oxide nanoparticles synthesized by *Penicillium commune* were described as biocompatible [37], carbon quantum dots derived from starch were associated with low toxicity [43], and glutathione-decorated magnetic nanoparticles [27] were reported as non-toxic, accessible, and retrievable. A more rigorous assessment was observed in the study on cellulose-derived nanomaterials, where the toxicity characteristic leaching procedure was used to evaluate the stability of adsorbed uranium [45]. However, these examples remain limited, and most studies did not include standardized ecotoxicity assays, long-term leaching tests, or evaluations of nanoparticle release after treatment.

Another critical issue concerns the recovery and management of spent nanosorbents. If nanoparticles are not efficiently separated from treated water, their release may introduce a secondary contamination pathway. Magnetic nanomaterials, such as Fe_3_O_4_-based composites and nZVI-supported systems, offer an important practical advantage because they can potentially be recovered using external magnetic fields, facilitating reuse and reducing the probability of uncontrolled nanoparticle dispersion [22]. Nevertheless, recovery efficiency, regeneration stability, and the fate of radionuclide-loaded materials are not systematically evaluated across the reviewed studies. There are some adsorption–desorption tests but are often limited to a small number of cycles and rarely address the long-term structural stability of the material or the safe disposal of the radionuclide-bearing adsorbent.

Scale-up also remains a major challenge. Although many studies report high removal efficiencies under controlled laboratory conditions, most experiments were performed in batch systems using synthetic waters and optimized operating conditions. Practical implementation would require validation in complex matrices, such as mine water, groundwater, or industrial effluents, where competing ions, organic matter, pH variability, and suspended solids may influence performance. Moreover, large-scale production of green nanoparticles must consider reproducibility, precursor variability, energy consumption, and the costs associated with recovery, regeneration, and final disposal.

Overall, while the “green” label is generally justified by the synthesis methods reported in the literature, the actual environmental and toxicological footprint of these nanomaterials remains largely unquantified. Future studies should therefore combine removal performance with standardized ecotoxicological testing, life-cycle assessment, nanoparticle release monitoring, regeneration studies, and end-of-life management strategies. This is essential to determine whether green-synthesized nanoparticles can move beyond promising laboratory-scale adsorbents and become environmentally safe and practically viable technologies for U and Th remediation.

### 4.5. Limitations of Included Studies

In general, most studies were lab-based, lacked long-term assessments, and used non-standardized protocols.

The most significant limitation is the substantial heterogeneity across studies, reflected in the diverse nanomaterials and agents used in green synthesis methods. However, more importantly, it is due to the lack of standardized evaluation methods. The studies were carried out over different pH ranges, contaminant concentrations, and adsorbent amounts. Thus, a comparison of the different materials in terms of performance is not feasible.

Moreover, it can be observed that most of the articles included were lab-based, in vitro investigations. Although many studies demonstrated high removal efficiency, these were often achieved using synthetic “clean” water. Only a minority of studies have validated their materials in real-world samples, such as mine water, groundwater, or industrial effluent, leaving a significant gap in understanding how these adsorbents perform in complex environmental matrices containing organic matter and multiple competing ions.

Furthermore, a lack of long-term evaluation is identified. Reusability tests were sometimes confined to a few cycles (i.e., 3–5) of adsorption and subsequent desorption. However, this may not be a good measure of how long these materials can last. Ultimately, as discussed previously, there is a lack of ecotoxicological information and life cycle assessments. Therefore, how safe and sustainable these “green” nanomaterials are in the long term is still unknown.

### 4.6. Implications for Future Research

The findings and limitations delineated in this review indicate several imperative avenues for future research. First, it is essential to close the gap between lab success and real-world performance. Future research should transition from small-scale batch experiments to upscaled processes, including tests in complex water matrices such as real mine water, groundwater or industrial effluents, preferably under continuous-flow conditions. Particular attention should be given to mixed-ion systems, since coexisting species may compete with U(VI) and Th(IV) for active adsorption sites or alter radionuclide speciation. Therefore, future studies should systematically evaluate the influence of competing ions and natural organic matter under environmentally relevant conditions. In addition, more standardized testing and reporting protocols are needed to improve comparability among studies. At minimum, future adsorption studies should clearly report the initial radionuclide concentration, adsorbent dosage, solution pH, ionic strength, temperature, contact time, water matrix composition, equilibrium concentration, regeneration conditions, and number of reuse cycles. Besides removal efficiency and maximum adsorption capacity, quantitative indicators such as the distribution coefficient (Kd) should also be reported, as they provide a normalized measure of adsorbent affinity and may allow more reliable comparisons between materials tested under different experimental conditions.

Additionally, a major gap in the current literature is that the “green” label is mainly based on the synthesis method and needs to be thoroughly verified. Comprehensive life cycle assessments (LCAs) are very important; therefore, future research should focus on measuring the long-term environmental impacts, potential ecotoxicity, and biodegradability of these nanomaterials after use.

Finally, the creation of hybrid systems is a promising new way to innovate. Instead of just using adsorption, the focus should be on combining these green nanoparticle adsorbents with other treatment methods, such as membrane filtration or photocatalysis. These multifunctional systems could adsorb radionuclides and degrade other organic pollutants simultaneously, making water treatment more effective and complete.

Beyond scientific and technical challenges, the transition of green nanotechnologies from laboratory studies to real-world applications also depends on regulatory frameworks, interdisciplinary collaboration, and solid evidence of its cost-effectiveness under operational conditions. Current research is still largely focused on material development and small-scale batch experiments, while fewer studies address process design, site-specific implementation, and end-of-life management of spent adsorbents. Future progress will require closer integration between materials scientists, process engineers, environmental specialists, and economists.

Regulatory support is the tightest bottleneck. These materials face a double hurdle: they are nanomaterials and treat radionuclides, both under strict oversight, and a “green” label does not replace safety data, so clear rules are needed for classification, handling, transport, and disposal of spent nanosorbents.

Finally, alongside regulatory approval, the most significant barrier to adoption is the need for clear cost-effectiveness evidence. While the high performance identified in this review is encouraging, these green technologies must ultimately compete with conventional options (such as ion exchange or precipitation) that have well-established operational costs. The primary economic advantage of the approaches reviewed is their use of low-cost precursors, such as waste biomass and agricultural byproducts. However, this initial advantage must be weighed against the total life-cycle cost, which includes reagents for functionalization, energy for regeneration, and, critically, the logistics and costs associated with solids handling and final disposal. Therefore, ideally, future studies must move beyond performance metrics to provide comprehensive techno-economic analyses and life-cycle costing.

## 5. Conclusions and Future Perspectives

The contamination of water sources with radionuclides such as uranium and thorium may pose a significant risk to the environment and human health. Conventional remediation methods, such as chemical precipitation or ion exchange, are notably limited by high implementation costs, reliance on toxic reagents, and the creation of secondary, polluting waste streams. Continuing to rely solely on traditional remediation techniques fails to account for solutions that are sustainable, cost-effective, and acceptable to those affected by contaminated environments (whether water or soil). Green synthesis of nanoparticles emerges as a potential alternative, offering a straightforward, eco-friendly approach to synthesize adsorbents with promising adsorption performance. It is well acknowledged that the transition to a circular economy needs to be accelerated. The research reviewed in this work demonstrates that “waste-to-resource” pathways are not just theory but a practical reality for creating high-performance materials. This paper shows that green-synthesized nanoparticles exhibit promising performance under laboratory-scale conditions for the remediation of radionuclide contamination. It was found that materials synthesized from agricultural residues, such as rice husks, or common plant extracts can achieve removal efficiencies consistently exceeding 90% and, in several cases, high maximum adsorption capacities under specific experimental conditions.

However, this pivotal research currently stands at a critical juncture. The “green” label, at present, is justified almost entirely by the synthesis method alone; most studies are confined to controlled laboratory settings using “clean” synthetic water, and there is a severe lack of life cycle assessments and ecotoxicological data to accurately verify the long-term environmental safety and footprint of these materials post-application. For this technology to fulfil its potential and move from lab experiments to global application, these gaps must be addressed. Future research must shift from batch experiments to scaling, from synthetic water to industrial effluents, and from assumed safety to verified, long-term environmental compatibility.

## Data Availability

This study is a literature review and does not involve generating or analysing new primary data. All data and references used in this review are available in the cited publications in the References section. No additional datasets were created or analysed in this work.

## Supplementary Materials

The following supporting information can be downloaded at: https://www.mdpi.com/article/10.3390/nano16130807/s1. PRISMA checklist.

## Abbreviations

The following abbreviations are used in this manuscript:

BET	Brunauer–Emmett–Teller
FTIR	Fourier-Transform Infrared Spectroscopy
LCA	Life Cycle Assessment
MOFs	Metal–Organic Frameworks
NORM	Naturally Occurring Radioactive Materials
NPs	Nanoparticles
NR	Not Reported
nZVCu	Nano Zerovalent Copper
nZVI	Nano Zerovalent Iron
PRISMA	Preferred Reporting Items for Systematic Reviews and Meta-Analyses
REEs	Rare Earth Elements
RoB	Risk of Bias
TEM	Transmission Electron Microscopy
Th	Thorium
U	Uranium
WHO	World Health Organization
ZIFs	Zeolitic Imidazolate Frameworks
